# Parental care compromises feeding in the pumpkinseed (*Lepomis gibbosus*)

**DOI:** 10.1007/s00114-018-1554-0

**Published:** 2018-03-26

**Authors:** G. Zięba, M. Dukowska, M. Przybylski, M. G. Fox, C. Smith

**Affiliations:** 1 Department of Ecology and Vertebrate Zoology, University of Łódź, Banacha 12/16, 90-237 Łódź, Poland; 2 Environmental and Life Sciences Graduate Program, Trent University, Peterborough, ON K9J 7B8 Canada; 3 Institute of Vertebrate Biology, Academy of Sciences of the Czech Republic, Květná 8, 603 65 Brno, Czech Republic; 4 School of Biology and Bell-Pettigrew Museum of Natural History, University of St Andrews, St Andrews, KY16 8LB UK

**Keywords:** Alternative mating strategy, Bayesian inference, Filial cannibalism, Male mating polymorphism, Parental care, Territoriality

## Abstract

Providing parental care is potentially costly. Costs can arise through elevated energy expenditure or from an increased risk of mortality. A cost of parental care can also occur because a parent is compromised in their ability to forage. We used pumpkinseed *Lepomis gibbosus*, a fish with an alternative male mating strategy, to test whether parental males differed in their feeding in comparison with females and cuckolder males. To address this question, we examined the stomach contents of female, cuckolder male, and parental male pumpkinseed during the breeding season over an entire diel cycle. We showed that parental males had a lower total weight of food in their stomachs in comparison with females, while cuckolder males did not. Parental males also had a lower weight and number of chironomids in their stomachs. The temporal pattern of feeding of parental males diverged from that of females, and they had a lower probability of pupal chironomids in their stomachs, which implies spatial segregation in foraging. Parental males had a greater probability of conspecific eggs in their stomachs than females, while the probability of egg cannibalism did not differ between cuckolder males and females. Overall, these finding meet predictions in accordance with an assumption that parental care and territoriality can compromise feeding.

## Introduction

Parental care can be costly, potentially constraining future reproductive success (Clutton-Brock 1991; Smiseth et al. 2012). Parental costs can compromise parental survival, for example by making a parent more susceptible to predators. Care may also increase physiological costs through elevated energy expenditure or if parental care curtails normal feeding activities (Clutton-Brock 1991; Klug et al. 2012).

Parental care is widespread in teleost fishes, with approximately one quarter of all teleost families including taxa that show some form of parental care (Wootton and Smith 2015). Paternal care is the most widespread form of care, which probably relates to male certainty of paternity associated with external fertilisation (Mank et al. 2005). The most common form of care is attending or guarding the offspring, whereby the parent protects the eggs and embryos from predators, including conspecifics (Wootton and Smith 2015). Guarding behaviour, which is often associated with territoriality, can be energetically expensive (e.g. Feldmeth 1983). Guarding the offspring may also severely limit normal foraging activities, though this has rarely been demonstrated (Wootton and Smith 2015).

The combination of elevated energy expenditure on parental care, along with restrictions placed on foraging, may leave a parent with an energy deficit that could compromise future reproductive opportunities. Rohwer (1978) proposed that parental males may engage in filial cannibalism (i.e. the consumption of guarded eggs and embryos) to make up this energy shortfall. Under this hypothesis, males effectively parasitize female reproductive investment to provide the energy necessary to complete care of the surviving offspring, thereby maximising their own fitness. An expectation is that poorly provisioned males should be more likely to engage in filial cannibalism than well-fed males (Smith 1992). Several studies support this prediction (reviewed in Wootton and Smith 2015), though others directly contradict Rohwer’s (1978) hypothesis, instead showing a positive relationship between filial cannibalism and egg density, and implicating a role for filial cannibalism as a behavioural mechanism for limiting density-dependent egg mortality (Payne et al. 2002; Klug and St Mary 2005; Klug et al. 2006).

Here, we investigate restrictions placed on foraging by parental male pumpkinseed *Lepomis gibbosus* (Centrarchidae). Parental care is performed exclusively by parental males in this species, which construct small, circular nests on the substrate. Females actively select males and deposit a clutch of between 1500 and 7000 eggs in the nest, spawning with multiple males over the course of a breeding season (Jordan et al. 2009). Parental care by the male comprises aggressive nest guarding and ‘fanning’, whereby the male aerates the eggs and embryos by driving a current of water over them with his pectoral fins. Pumpkinseed usually spawn from late spring to early summer at temperatures between 15 and 25 °C (Jordan et al. 2009). Once eggs are deposited in the nest, the male confines his activities to the immediate vicinity of the nest to protect them from predators and provide care, which is energetically costly (Cooke et al. 2006) and can result in weight loss and increased mortality rates over the breeding period (Rios-Cardenas and Webster 2005). Spawning bouts typically last 10–11 days and are repeated several times within a spawning season (Scott and Crossman 1973).

A striking feature of the pumpkinseed mating system is the existence of an alternative male mating strategy, termed a ‘cuckold’ (Gross 1979, 1982). Cuckold males attempt to fertilise the eggs deposited by females in the nests of parental males and thereby avoid the costs associated with nest preparation, territoriality and parental care (Taborsky 2008). Cuckold males are small, passive and typically swim close to the substratum and between nest borders (Gross 1982). They participate in matings between females and parental males, competing effectively with parental males through sperm competition. Because they express no parental care or territoriality, they experience no constraints on foraging associated with these behaviours.

Pumpkinseed are omnivorous, demonstrating high trophic and/or resource polymorphism (Copp et al. 2004), foraging mainly at dawn and dusk (Collins and Hinch 1993; Johnson and Dropkin 1993) and taking advantage of the full range of available prey (Godinho et al. 1997). There appear to be distinct temporal patterns of feeding associated with parental care in pumpkinseed. In spring, following a rise in water temperature, both growth and food consumption rates increase (Rios-Cardenas 2005). However, feeding during the nesting period in June and July appears to decrease (Thorp et al. 1989; Collins and Hinch 1993), which may be a function of parental males foraging in shallow water around their nests where food resources are limited (Thorp et al. 1989; Rios-Cardenas and Webster 2005). Parental males may also vary the times at which they feed over the diel cycle (Persson 1983), a consequence of differences in the availability and accessibility of specific prey types (Keast and Welsh 1968), reflecting changes in the activity and vulnerability of prey (Boujard and Leatherland 1992).

To date, most work on the dietary habits of pumpkinseed have focused on ontogenetic dietary shifts (e.g. García-Berthou and Moreno-Amich 2000; Maazouzi et al. 2010), differences in diet in contrasting environments (e.g. Almeida et al. 2009), diet partitioning in competition with other fish taxa (e.g. Wolfram-Wais et al. 1999; Declerck et al. 2002), or trophic polymorphism as a consequence of differential use of available resources (e.g. Mittelbach et al. 1999; Yavno et al. 2014). However, no study has yet addressed the qualitative and quantitative changes in diet in pumpkinseed in relation to sex and mating strategy.

Here, we examine the diet of pumpkinseed in relation to sex and mating strategy over a complete diel cycle during the peak of breeding. We tested the prediction that parental males are restricted in foraging while performing parental care, expressed as a quantitative and qualitative difference in diet in comparison with females and cuckold males, neither of which face restrictions in foraging as a result of nest guarding.

Diet studies on pumpkinseed have tended to employ low resolution taxonomic identification of prey species (Wolfram-Wais et al. 1999), which can generate overestimates of diet overlap among groups (Rezsu and Specziár 2006). We followed the recommendation of Wolfram-Wais et al. (1999) and employed a detailed taxonomic resolution of prey taxa in the diet of pumpkinseed to enable us differentiate feeding microhabitats.

We predicted that (1) females and cuckolder males would have a greater mass and a higher number of prey items in their stomachs than parental males; (2) parental males would have a higher proportion of food items foraged from the water column than females and cuckolder males; and (3) parental males would have a greater probability of containing conspecific eggs in their stomachs than females and cuckolder males.

## Materials and methods

### Study site

The study was conducted in north-western Poland on 6th of July 2013, at the peak of spawning, in Brodowski Pond, a former clay pit in the city of Szczecin (latitude 53.450138, longitude 14.565363). Brodowski Pond is approximately 0.92 km^2^ with a maximum depth of 7 m. Water temperature reflects natural diurnal fluctuations in air temperature, varying from 4 °C in winter under thick ice cover, to 25 °C in summer. At midday on the day of sample collection, water temperature was 22.4 °C, water conductivity 1345 μS cm^−1^ and dissolved oxygen concentration 9.83 mg O_2_ l^−1^. Sunrise on the day of sampling was at 04.42 and sunset was at 21.31. Pond banks were strengthened with fascine and boulders and were overgrown with common reed (*Phragmites australis*). The bottom substrate was dominated by sand and clay. Water transparency exceeded 1 m and dense patches of submerged vegetation (mainly *Elodea canadensis*) occurred in the margins. Pumpkinseed nests were observable along the shallow, sandy edges of the pond on the day of sampling. The original source population of pumpkinseed in the pond was not known.

### Sample collection and processing

To estimate fish diet quantity and quality, 123 sexually mature pumpkinseeds were collected from the pond margin by electrofishing. Groups of 15–17 fish were collected at 3-h intervals over a 24-h period: at 03.00, 06.00, 09.00, 12.00, 15.00, 18.00, 21.00 and 00.00. To minimise disturbance, electrofishing was conducted from the pond bank with a backpack electroshocker (EFGI 650, BSE Bretschneider Specialelektronik, Germany), with power output set to immobilise small fish with minimal stress and injury. On each sampling occasion, a different section of pond margin was sampled to avoid capturing males that had occupied the nest sites of parental males removed during earlier collections. A target number of 15 fish was collected in each sampling period, which typically took approximately 5 min. to accomplish and yielded the desired balance of fish sexes and mating strategies (Table 1). Approximately 10% of the total pond margin was sampled over the entire 24-h period.

**Table 1:** Distribution of parental male, cuckolder male and female pumpkinseed *Lepomis gibbosus* among samples collected over a 24-h period

Time	Female	Cuckolder	Parental
03.00	6	1	8
06.00	6	5	4
09.00	7	2	8
12.00	7	4	4
15.00	5	3	8
18.00	10	4	1
21.00	8	4	3
00.00	7	5	3

After capture, fish were immediately euthanized with an overdose of clove oil and preserved in 4% buffered formalin. Specimens were later measured for total length (TL, nearest mm), weighed (nearest 10 mg) and sexed. Stomach contents were dissected and weighed (nearest 1 mg) and preserved on microscope slides in glycerine. Food items were subsequently counted and identified to the lowest possible taxonomic level (ranging from species to order) under a dissecting stereomicroscope (Nikon SMZ1000, Japan). Male reproductive strategy (either parental or cuckolder) was identified based on the gonado-somatic index (GSI; Wootton 1998) by fitting normal frequency distributions to GSI data using the Bhattacharya method (Bhattacharya 1967). A threshold GSI value of 3.2 was taken as the intercept separating normal distributions, with specimens with GSI values below the threshold categorised as parentals and those with GSI values equal to or above 3.2 as cuckolders (see Zięba et al. 2018). Based on these criteria, 39 nesting males, 28 cuckolders and 56 females were collected overall, with each sex/strategy represented in each sampling period (Table 1). While we did not know which males assigned to the parental group were actively parenting at the time of capture, we made efforts to ensure it was predominantly males engaged in parental care that were targeted. Thus, the study corresponded with the peak in spawning for this population, which we identified by regular site visits and visual inspection of nest building and territorial behaviour. Immediately prior to sampling, we also visually assessed areas of the pond with the greatest density of nests (obvious as patches of cleared detritus and vegetation) and with males actively engaged in territorial contests with neighbours. These sites were subsequently the focus of sampling. Finally, parental male pumpkinseed tend to stay close to their nest, even when disturbed, further reinforcing the likelihood that is was males actively engaged in parental care that were primarily, if not exclusively, represented in samples.

The study was carried out under a licence from the Local Ethical Committee for Experiments on Animals in Łódź (No 44/ŁB 561/2011), accompanied by the necessary licences for conducting electrofishing survey at the study site.

### Data analysis

Initial analyses of food consumption showed evidence of autocorrelation of residuals with time of day. To accommodate temporal dependency in the data we used R (version 3.4.2; R Development Core Team 2017) to fit all models in a Bayesian framework using Integrated Nested Laplace Approximation (R-INLA; Rue et al. 2017). The total weight of food in the stomachs of pumpkinseed was modelled using a random walk (RW2) trend model fitted to data from each time period. For each time period (*t* = 1, ..., 8), we modelled diet weight for fish *i* at time period *t* as follows:

$$ {TW}_{it}\sim N\left({\mu}_{it},{\sigma}^2\right) $$

$$ E\left({TW}_{it}\right)={\mu}_i\;\mathrm{and}\ \mathit{\operatorname{var}}\left({TW}_{it}\right)={\sigma}^2 $$

$$ {\mu}_{it}={\beta}_1+{\beta}_2\times {TL}_{it}+{\beta}_3\times {strategy}_{it}+{time}_i $$

$$ {time}_t\sim N\left(0,{\sigma}_{time}^2\right) $$

where *TW*_*it*_ is the weight of all food items in the stomach of fish *i* in time period *t*. The variable *strategy*_*it*_ is a categorical covariate with three levels, corresponding with fish mating strategy; female, cuckolder or parental. The model also contained a linear effect for fish total length (*TL*_*it*_). The same approach was used to model weight of chironomids in the stomach, which were the largest component in the diet. For the number of chironomids in the stomach *NC*_*it*_ for fish *i* in time period *t*, the data were modelled using a Poisson model, which took the form:

$$ {\displaystyle \begin{array}{c}{NC}_{it}\sim Poisson\left({\mu}_{it}\right)\\ {}E\left({NC}_{it}\right)={\mu}_{it}\\ {}\log \left({\mu}_{it}\right)={\eta}_{it}\\ {}{\eta}_{it}={\beta}_1+{\beta}_2\times {TL}_{it}+{\beta}_3\times {strategy}_{it}+{time}_i\\ {}{time}_t\sim N\left(0,{\sigma}_{time}^2\right)\end{array}} $$

We fitted a Bernoulli model to data on of the presence of pupal chironomids, which occur in the water column, using the following:

$$ {\displaystyle \begin{array}{c}{PC}_{it}\sim Binomial\left({\pi}_{it}\right)\\ {}E\left({PC}_{it}\right)={\pi}_{it}\\ {}\mathrm{logit}\left({\pi}_{it}\right)={\eta}_{it}\\ {}{\eta}_{it}={\beta}_1+{\beta}_2\times {TL}_{it}+{\beta}_3\times {strategy}_{it}+{time}_i\\ {}{time}_t\sim N\left(0,{\sigma}_{time}^2\right)\end{array}} $$

where *PC*_*it*_ is the probability of pupal chironomids occurring in the stomach of fish *i* in time period *t*. The same approach was used to model probability of egg cannibalism.

## Results

**Fig. 1:**
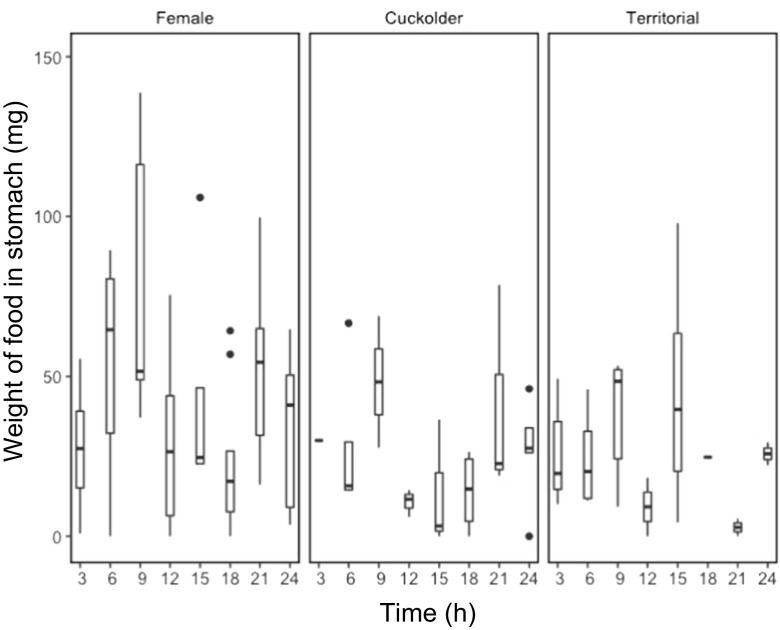
Boxplot showing the total weight of food items in the stomachs of female, cuckolder male and parental male pumpkinseed *Lepomis gibbosus* in each of eight sampling periods over a complete diel cycle

**Fig. 2:**
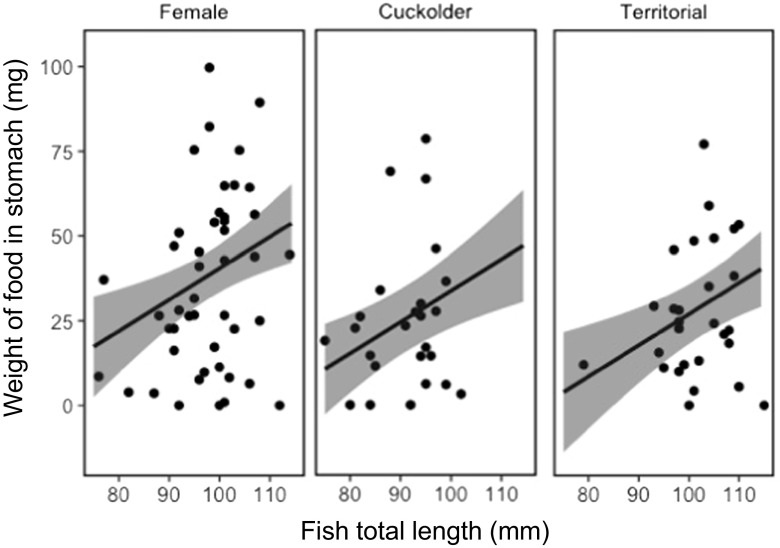
Mean fitted weight (solid line) and 95% credible intervals (shaded area) of the total weight (mg) of food items in the stomachs of female, cuckolder male and parental male pumpkinseed *Lepomis gibbosus* against fish total length (mm) over a full diel cycle

**Fig. 3:**
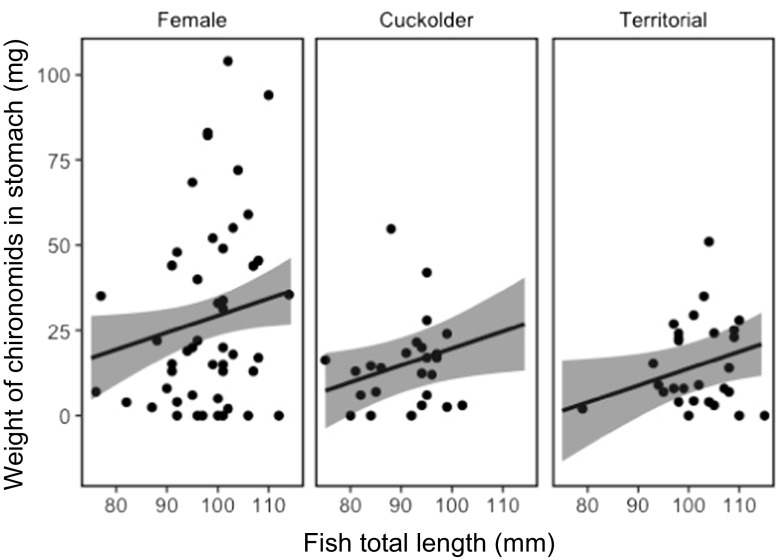
Mean fitted weight (solid line) and 95% credible intervals (shaded area) of the weight (mg) of chironomids in the stomachs of female, cuckolder male and parental male pumpkinseed *Lepomis gibbosus* against fish total length (mm) over a full diel cycle

**Fig. 4:**
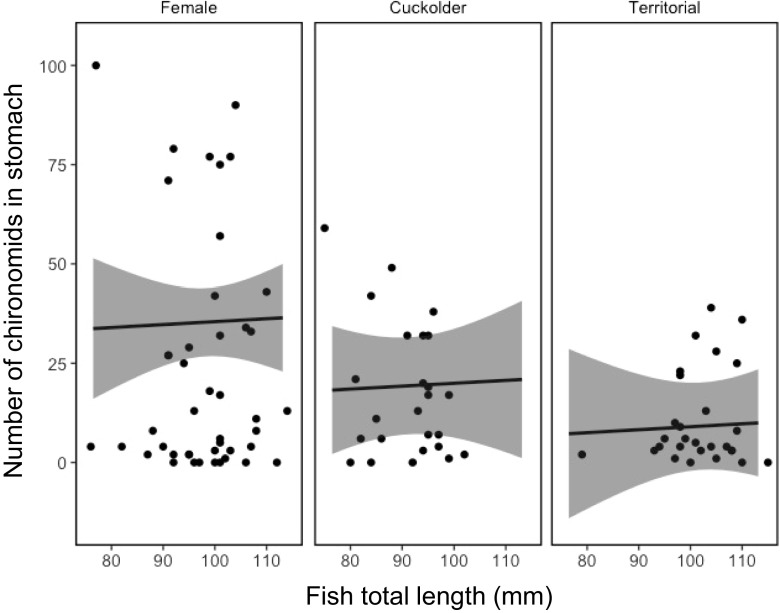
Mean fitted number (solid line) and 95% credible intervals (shaded area) of the number of chironomids in the stomachs of female, cuckolder male and parental male pumpkinseed *Lepomis gibbosus* against fish total length (mm) over a full diel cycle

Total weight of food consumed by pumpkinseed showed strong temporal dependency, varying with mating strategy (Fig. 1). There was a statistically important difference in the total weight of food in the stomachs of parental males in comparison with that of females (Table 2), though not between cuckolder males and females (Table 2), with parental males having less food in their stomachs (Fig. 2). Chironomids were the largest component of the diet of pumpkinseed in the study, comprising 52% of the diet by weight. There was a lower weight (Table 3; Fig. 3) and number (Table 4; Fig. 4) of chironomids in the stomachs of parental males in comparison with that of females. There was no difference between cuckolder males and females in the weight of chironomids in their stomachs (Table 3), though there was a lower number of chironomids in the stomachs of parental males (Table 4; Fig. 4).

**Table 2:** Posterior mean estimates for total weight of food in the stomachs of pumpkinseed *Lepomis gibbosus* as a function of total length and mating tactic, modelled using a Gaussian random walk trend model. CrI is the 95% Bayesian credible interval. Credible intervalsthat do not contain zero in italics indicate a statistically important difference betweenfemales and males with the tactic indicated

Model parameter	Posterior mean	Lower CrI	Upper CrI
Intercept_(female)_	0.28	*0.01*	*0.54*
Total length	0.28	*0.08*	*0.48*
Tactic_(cuckolder)_	− 0.38	− 0.86	0.10
Tactic_(parental)_	− 0.63	*− 1.06*	*− 0.20*

**Table 3:** Posterior mean estimates for weight of chironomids in the stomachs of pumpkinseed *Lepomis gibbosus* as a function of total length and mating tactic, modelled using a Gaussian random walk trend model. CrI is the 95% Bayesian credible interval. Credible intervalsthat do not contain zero in italics to indicate statistical importance

Model parameter	Posterior mean	Lower CrI	Upper CrI
Intercept_(female)_	0.33	*0.07*	*0.59*
Total length	0.28	*0.07*	*0.47*
Tactic_(cuckolder)_	− 0.42	− 0.89	0.06
Tactic_(parental)_	− 0.79	*− 1.22*	*− 0.37*

**Table 4:** Posterior mean estimates for number of chironomids in the stomachs of pumpkinseed *Lepomis gibbosus* as a function of total length and mating tactic, modelled using a Poisson random walk trend model. CrI is the 95% Bayesian credible interval. Credible intervalsthat do not contain zero in italics to indicate statistical importance

Model parameter	Posterior mean	Lower CrI	Upper CrI
Intercept_(female)_	3.00	*2.54*	*3.46*
Total length	0.004	− 0.004	0.009
Tactic_(cuckolder)_	− 0.65	*− 0.76*	*− 0.54*
Tactic_(parental)_	− 1.25	*− 1.37*	*− 1.13*

**Fig. 5:**
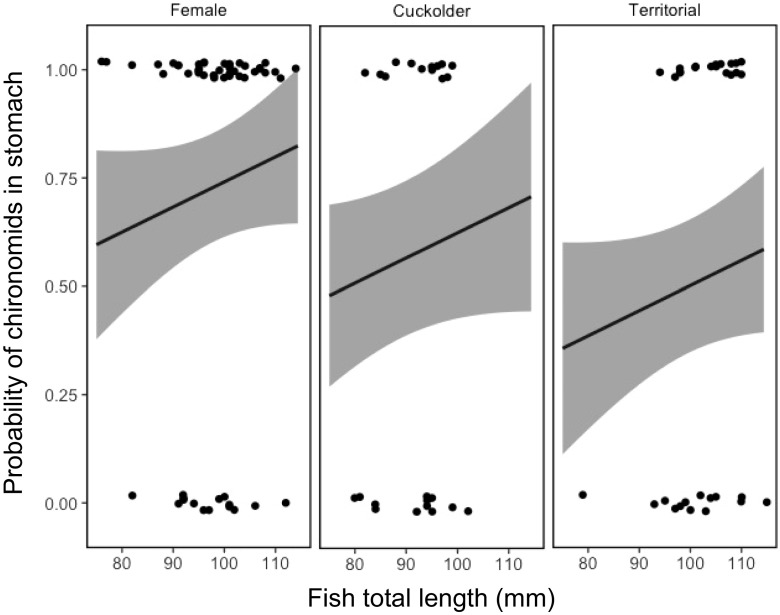
Mean fitted probability (solid line) and 95% credible intervals (shaded area) of the occurrence of chironomid pupae in the stomachs of female, cuckolder male and parental male pumpkinseed *Lepomis gibbosus* against fish total length (mm) over a full diel cycle

**Fig. 6:**
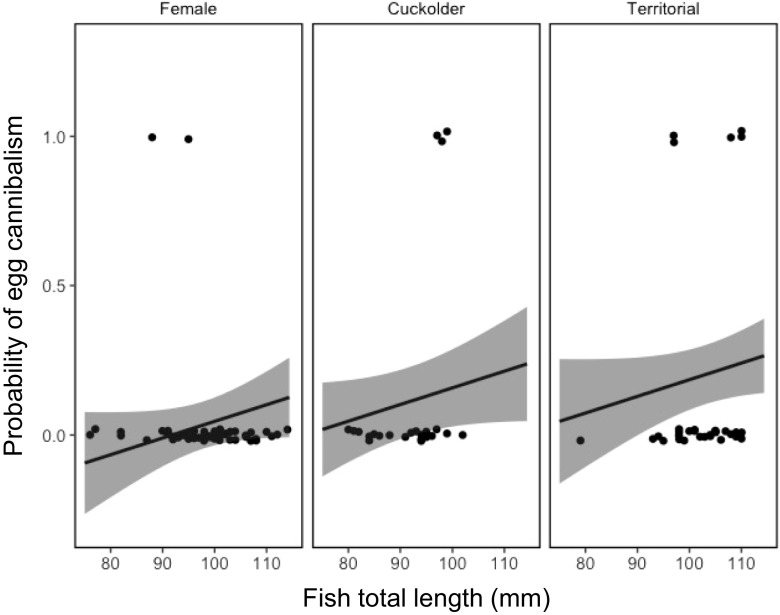
Mean fitted probability (solid line) and 95% credible intervals (shaded area) of the occurrence of conspecific eggs in the stomachs of female, cuckolder male and parental male pumpkinseed *Lepomis gibbosus* against fish total length (mm) over a full diel cycle

There was also a statistically important lower probability of chironomid pupae, which occur in the water column, occurring in the stomachs of parental males in comparison with females, though not between cuckolder males and females (Table 5; Fig. 5). However, there was a statistically important greater probability of conspecific eggs in the stomachs of parental males than females, but no difference between females and cuckolder males (Table 6; Fig. 6).

**Table 5:** Posterior mean estimates for the probability of pupal chironomids occurring in the stomachs of pumpkinseed *Lepomis gibbosus* as a function of total length and mating tactic, modelled using a Bernoulli random walk trend model. CrI is the 95% Bayesian credibleinterval. Credible intervals that do not contain zero in italics to indicate statisticalimportance

Model parameter	Posterior mean	Lower CrI	Upper CrI
Intercept_(female)_	− 1.59	− 5.24	1.80
Total length	0.03	− 0.01	0.01
Tactic_(cuckolder)_	− 0.53	− 1.54	0.48
Tactic_(parental)_	− 1.09	*− 2.00*	*− 0.21*

**Table 6:** Posterior mean estimates for the probability of conspecific eggs occurring in the stomachs of pumpkinseed *Lepomis gibbosus* as a function of total length and mating tactic, modelled using a Bernoulli random walk trend model. CrI is the 95% Bayesian credible interval.Credible intervals that do not contain zero in italics to indicate statistical importance

Model parameter	Posterior mean	Lower CrI	Upper CrI
Intercept_(female)_	− 11.09	− 21.04	− 2.49
Total length	0.08	− 0.01	0.17
Tactic_(cuckolder)_	1.87	− 0.07	3.98
Tactic_(parental)_	1.65	*0.05*	*3.51*

## Discussion

The constraints imposed by parental care, particularly when associated with territorial defence of a nest site, are assumed to limit the ability of a parent to forage (Clutton-Brock 1991). Though a generally held assumption, few studies have directly tested the extent to which parents are constrained in their ability to feed. Here, we demonstrate that parental male pumpkinseed had a lower total weight of food in their stomachs in comparison with females. Parental males also had a lower weight and number of chironomids in their stomachs; the principal constituent of their diet (e.g. Godinho et al. 1997; Wolfram-Wais et al. 1999; Declerck et al. 2002; Nikolova et al. 2008), and a lower probability of pupal chironomids, which occur in the water column. Notably cuckolder males, which engage in matings like parental males, but without the constraints imposed by parental care or territorial defence of a nest, did not differ from females in their stomach contents. In contrast, the probability of pumpkinseed eggs in the stomach of parental males was higher than in females, while the probability of egg cannibalism did not differ between cuckolder males and females.

Restrictions placed on foraging represent a potentially important component of parental costs. The focus of studies on parental costs has been on physiological costs and time (opportunity) costs of lost matings (Clutton-Brock 1991; Smith and Wootton 1995; Alonso-Alvarez and Velando 2012). However, limitations on foraging associated with care and defence of the offspring are rarely quantified. The exceptions are fish species with oral brooding of eggs and young stages, which necessarily imposes an interruption to feeding. In the maternal mouthbrooding cichlid *Haplochromis argens*, females that performed parental care suffered a 61% reduction in body weight in comparison with control females, which had their eggs removed immediately after spawning and were able to feed (Smith and Wootton 1994). Similarly, in the biparental mouthbrooding mango tilapia (*Sarotherodon galilaeus*), females suffered a cost of reduced breeding frequency and clutch size (Balshine-Earn 1995). In the present study, males had lower stomach fullness over a diel cycle during the breeding phase, which implies they suffer a feeding deficit in comparison with cuckolder males and females. Parental males may also expend more energy in performing parental care. Egg fanning (e.g. Smith and Wootton 1999) and territorial defence (e.g. Feldmeth 1983) are associated with substantially elevated energy expenditure in teleosts. Thus, a combination of a constraint on feeding and increased energy expenditure in parental males may impose a reproductive cost. Whether parental male pumpkinseed suffer a true fitness cost, measurable in terms of future reproduction (sensu Williams 1966), was not demonstrated here, though our findings provide circumstantial evidence that such a cost may exist.

The pattern of stomach fullness shown by pumpkinseed over a diel cycle, irrespective of mating strategy, showed peaks at dawn and dusk (Fig. 1). This observation matches previous findings on the feeding behaviour of this species (Collins and Hinch 1993; Johnson and Dropkin 1993). Notably, the pattern of feeding was most pronounced for parental males, which appeared to feed predominantly at these times, in contrast with females that had a consistently high quantity of food in their stomachs, except at sampling periods at midnight and early morning. In addition to this temporal difference in stomach contents, the lower probability of pupal chironomids, which occur primarily in the water column (Armitage 1995), in the stomachs of parental males in comparison with females and cuckolder males implies a spatial segregation based on mating strategy. Because of their attachment to a territory and offspring, males may be forced to forage predominantly on the substrate close to their nest. Females and cuckolders do not face the same spatial limitations and instead appear to forage both on the substrate and in the water column.

A conspicuous feature of the life-history of the pumpkinseed is the expression of two starkly different mating strategies; parental male and cuckolder, a rare example of a male mating polymorphism (Gross 1982; Wootton and Smith 2015). The mechanism by which these mating polymorphisms are maintained in the same population is generally considered to be through negative frequency-dependent selection (Dodson et al. 2013). Under this assumption, the prediction is that the reproductive success of each mating strategy should vary inversely with the frequency of individuals that display the strategy. At the population level, each mating strategy is predicted to generate an equivalent fitness payoff (Shuster 2010). An alternative model is the status-dependent selection hypothesis of Gross (1996). Here, alternative mating strategy reflects differences in physical condition or social status among males, with one strategy linked to high condition or status, and the alternative to a lower condition or status. Under this model, the average fitness of individuals adopting the alternative strategy is expected to be lower, though there is a predicted switch point between mating strategies when fitness will be equivalent. While the average fitness of the two strategies is not equivalent, the prediction is that an individual will achieve its maximum fitness given its current condition or status. Our current understanding of mechanisms that underpin the evolution of male mating polymorphisms is incomplete.

In the present study, we show that, in addition to strikingly different modes of reproduction, patterns of feeding between male mating strategies in pumpkinseed also diverge. Because the pattern of growth in fishes is indeterminate, growth rate and body size are key intrinsic factors that dictate life-history evolution (Wootton 1998), with direct implications for male mating phenotype, which are largely determined by body size (Taborsky 2008). An implication of the feeding constraints on parental males that we show here is that selection will favour smaller and younger males to adopt the cuckolder strategy because of limitations placed on feeding imposed by parental care. For larger males, restrictions on feeding may be less likely to translate into a reproductive cost, because negative effects of restricted ration will tend to be buffered by a larger body size (Wootton 1998). In smaller males, however, the effects of food limitation may be substantial due to greater relative energy expenditure on parental care and territoriality, less effective protection of offspring and poorer subsequent success in mate acquisition. Thus, under the status-dependent selection hypothesis, any constraints on foraging imposed by territoriality and parental care would be predicted to favour the evolution of a male mating polymorphism that does not suffer a restriction on feeding, such as the cuckolder strategy. If the case, male mating polymorphisms might be expected to occur more frequently in teleosts with costly male care, whereas mating polymorphisms are relatively rare (Wootton and Smith 2015). However, other selective pressures, such as predation, may constrain the evolution of male mating strategies that select for small body size. Notably, in the related bluegill sunfish (*Lepomis macrochirus*), male size at maturation is responsive to resource availability (Aday et al. 2006), which implies that life-history evolution in these fishes may be sensitive to energetic constraints. The role of feeding limitation in the evolution of male mating polymorphisms, and particularly the cuckolder strategy in pumpkinseed, is one that warrants further research.

Cannibalism, including egg cannibalism, is common in teleosts (Smith and Reay 1991). Here we showed that parental males had a statistically important greater probability of having conspecific eggs in their stomachs. Rohwer (1978) proposed that parental males may engage in filial cannibalism to augment their diet, specifically in cases were males faced restrictions on foraging. Support for Rohwer (1978) is equivocal, with some studies purporting to show this effect, while others do not (see Smith 1992; Payne et al. 2002; Klug et al. 2012 for fuller discussion). Our results lend limited support to this hypothesis, though we were unable to demonstrate whether the eggs in the stomachs of parental males came from a males’ own nest or that of a neighbour, which is a critical assumption. However, our results at least highlight that parental males engage in egg cannibalism, either of their own or unrelated eggs, which may supplement their diet when facing restricted foraging opportunities while engaged in parental care.

A caveat to this study was that sampling occurred over a single diel period in one population. This approach reduced spatial and season temporal effects in the analysis but limits the generality of conclusions. Further comparable studies among population over the entire reproductive cycle of pumpkinseed would be informative.

In summary, observed differences in quantity and quality of prey in examined stomachs of female, cuckolder male and parental male pumpkinseed over a full diel cycle indicate the existence of spatio-temporal differences in the feeding pattern of sexually mature *L. gibbosus* over reproductive period. Within the limitations of the study, these findings are in accordance with an assumption that parental care and territoriality compromise feeding in pumpkinseed.
